# Lumbar Laminectomy Precedes Positional Headache and MRI-Confirmed Pseudomeningocele With Duro-Cutaneous Fistula

**DOI:** 10.7759/cureus.37946

**Published:** 2023-04-21

**Authors:** Bradley E Kasper, Ishan R Perera, Sydney E Moriarty, Frederic Rawlins

**Affiliations:** 1 Medical School, HOLO Labs - Simulation and Educational Technology, Edward Via College of Osteopathic Medicine, Blacksburg, USA; 2 Emergency Medicine, HOLO Labs - Simulation and Educational Technology, Edward Via College of Osteopathic Medicine, Blacksburg, USA

**Keywords:** post-laminectomy complication, pseudomeningocele (pm), neurologic imaging, incidental durotomy, lumbar, dural fistula

## Abstract

Pseudomeningoceles (PMs) are collections of cerebrospinal fluid (CSF) occurring as a direct result of a dural rent. This article presents a well-documented case of a 68-year-old male presenting to the emergency department with postoperative lumbar PM with a duro-cutaneous fistula. It was initially recognized on palpation of the patient’s postoperative incision site and later diagnosed with magnetic resonance imaging (MRI). Incidental durotomies (IDs) leading to PMs are a rare complication of laminectomies and other spinal surgeries. A thorough physical exam, diagnostic imaging, and lumbar drainage to survey the integrity of the dura mater are important aspects of postoperative care.

## Introduction

Pseudomeningoceles (PM) are extradural collections of cerebrospinal fluid (CSF) which form after the dura mater is torn [1]. These rare complications of spinal surgery occur after approximately 0.7% to 2% of laminectomies or discectomies [1]. Determining factors for PM formation include the size of the dural defect, strength of the surrounding musculature and structures, and pressure due to inflowing CSF [2]. In a trial following 553 patients after lumbar laminectomies, 70 patients experienced incidental durotomies (ID) - three of these cases presented with PMs [3]. This study also found the incidence of ID to be highest in re-operative surgeries following findings of PMs and nonspecific CSF leaks [3]. An increased incidence of IDs in re-operative cases is further supported in a separate review of 3183 lumbar surgeries [4]. These are rarely diagnosed in clinical practice, specifically during the immediate postoperative course, due to their commonly asymptomatic nature [1]. We present a rare case of a symptomatic PM initially recognized upon palpation of the postoperative incision site and diagnosed with lumbosacral magnetic resonance imaging (MRI). Our goal is to emphasize the importance of a thorough physical examination, postoperative imaging, and placement of a lumbar drainage device after spinal surgeries with concurrent IDs. 

## Case presentation

History and examination

**Figure 1:**
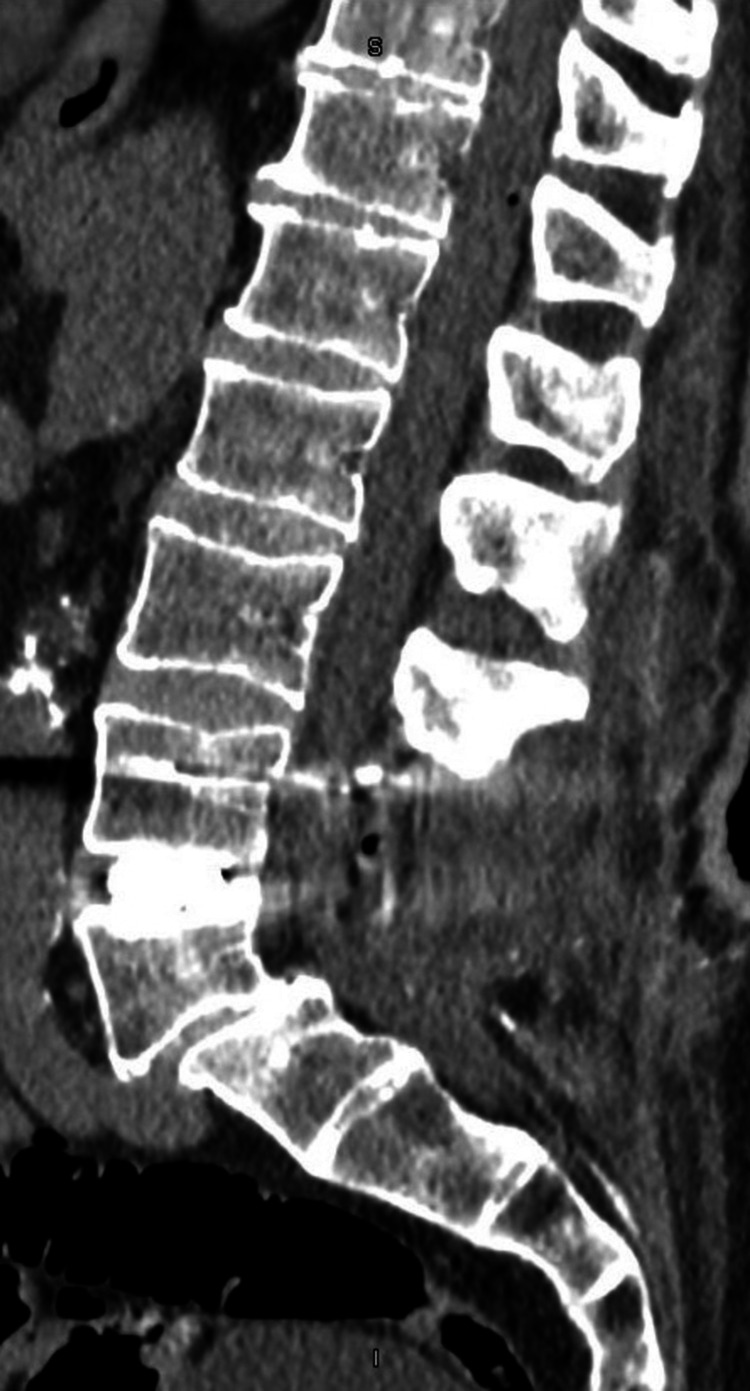
Postoperative sagittal CT scan shows new, larger laminectomies at L4 and L5 with proper placement of new disc prosthesis and new pedicle screws at L4 connected to previously placed pedicle screws at L5.

A 68-year-old male patient presented to the emergency department with a primary complaint of persistent post-operative bleeding. The patient had undergone uncomplicated spinal surgery three days prior, including large laminectomies at L4-5 with new disc prosthesis, concomitant posterior stabilization, and removal of S1 hardware. A lumbar CT without contrast was conducted and demonstrated a small amount of gas at L2/L3 and L4/L5 levels (Figure 1). 

No large postoperative fluid collection was noted, only nonspecific inflammation and stranding posteriorly at L4/L5 level. The patient was discharged with a diagnosis of post-laminectomy syndrome of the lumbar region. 

**Figure 2:**
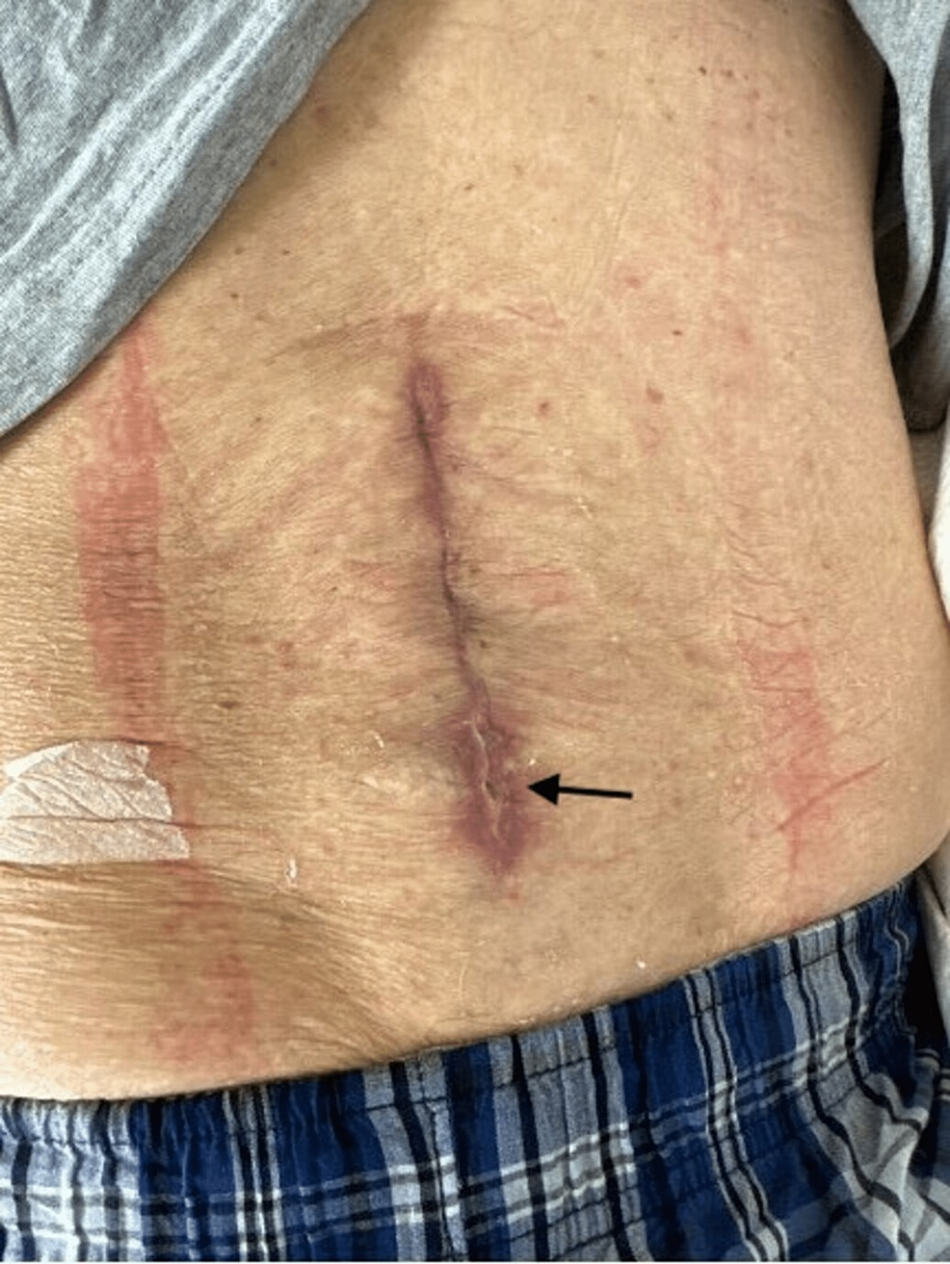
Arrow pointing to the site of CSF drainage. CSF: cerebrospinal fluid

Four weeks later, the patient presented to the emergency department with low back pain, bilateral lower limb numbness, a headache that was aggravated upon standing and alleviated by lying down, and persistent non-purulent drainage. The incision site was noted at 16 cm in length (Figure 2).

**Figure 3:**
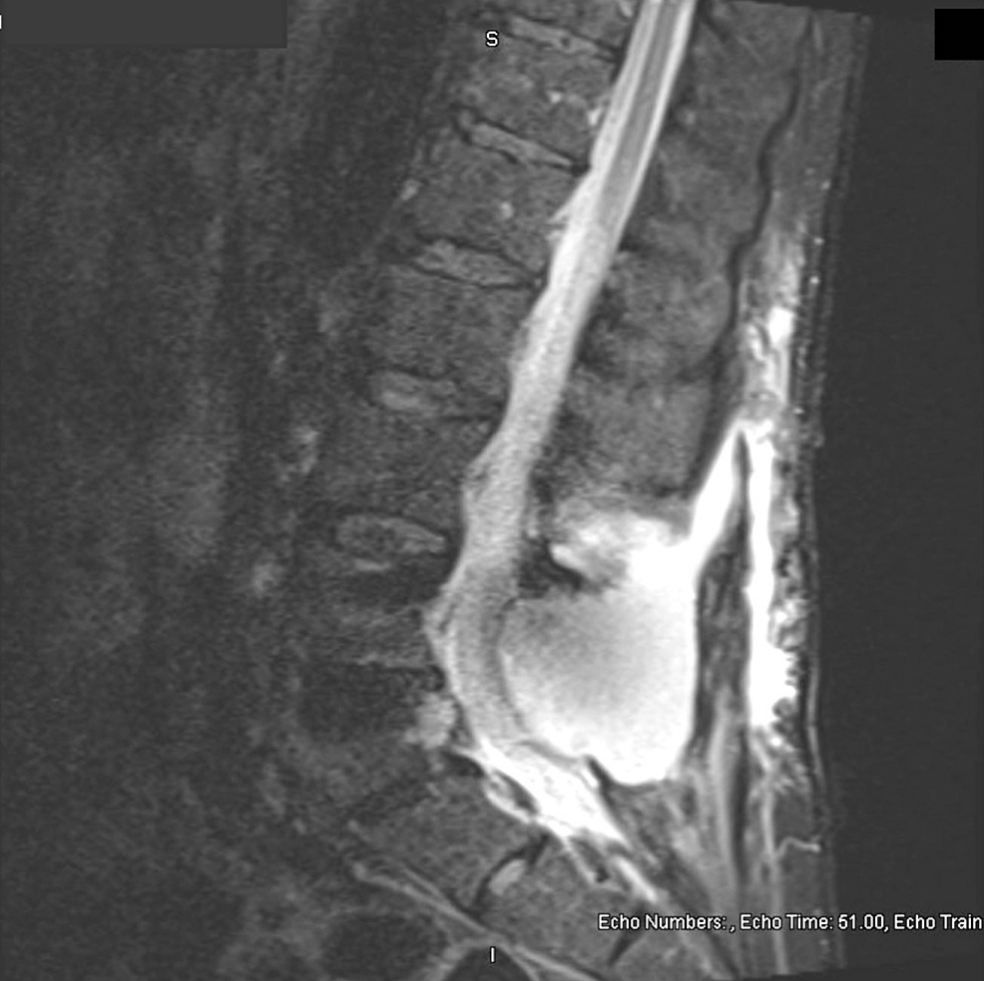
Large posterior mass with fistulous tract visible on sagittal lumbosacral T2-weighted MRI without contrast. Mass measures 5x8x9-cm (AP, transverse, and vertical, respectively).

Minor bleeding was observed without purulent discharge. Upon palpation, a clear serous drainage arose from the most caudal aspect of the incision site. The patient was otherwise well-developed and well-nourished. A lumbosacral MRI with and without contrast was conducted and demonstrated a large fluid collection (Figure 3).

The collection was located posterolateral to the spinal canal on the left, at sites of defect from the L4 and L5 laminectomies. Mass effect with lateral displacement of the thecal sac to the right, creating moderate to severe central stenosis at L4/L5 and L5/S1 levels was observed. The fluid collection continued caudally until reaching the inferior margin of the thecal sac at S1. A superior fistulous tract, showing minor peripheral enhancement, extended posteriorly through subcutaneous tissues to the skin’s surface inferiorly. 

Outcome

Based on the patient’s physical exam and imaging findings, neurosurgery was consulted. Neurosurgery found the patient neurologically intact and afebrile with no white count. The patient was discharged with instructions to follow up with neurosurgery and to continue Keflex (cephalosporin) and Percocet (oxycodone/paracetamol). The patient consented to surgical exploration and repair of the dural rent - the patient did not require further decompression. The procedure was performed without complication and achieved the desired therapeutic effect. 

## Discussion

Observations

PMs secondary to IDs are rare complications of lumbar laminectomies [1]. Collections of CSF can often be missed on the physical exam due to their propensity to present as asymptomatic or with nonspecific symptoms (nausea, vomiting, headaches, etc.) [1,3]. The delayed collection of fluid, physical examination findings of clear serous drainage from the incision site, and complaint of headaches upon standing with relief when lying down are features unique to this case. The physician’s thorough physical exam allowed the team to identify the CSF leak and MRI utilization was necessary to confirm its presence [3]. 

Lessons

As time progresses, technologies in the field of medicine progress as well. One such technology often used after spinal surgeries to decrease the formation of extradural collections of CSF, is the lumbar drain. The recommendation for placement of lumbar drains after spinal surgeries to counteract CSF accumulation from incidental dural tears was reinforced in a *Techniques in Orthopaedics* article in 2012 [5]. The use of such drains has demonstrated an over 90% success rate in the treatment of CSF fistulas by decompressing the subfascial space - providing a proper environment to heal the dural tear [5]. The increasing usage of postoperative lumbar drains should decrease the incidence of PMs with all other factors remaining unchanged. Application of lumbar drain to this case is postulated to have prevented the sequelae of the original laminectomy and fixation procedure. 

The most recent estimates of incidence for postoperative PMs, with sufficient power, are found in studies performed in 1947, 1975, and 1983 [1]. Due to changes in medical technologies and practices alone, further research in evaluating the incidence of PMs should be performed. 

## Conclusions

Our report provides an analysis of the etiology and presentation of PMs. Postoperative PMs typically present asymptomatic and are rarely diagnosed during the immediate postoperative period. However, our report identified a symptomatic PM, which was initially detected through physical examination and subsequently confirmed with a lumbosacral MRI. Our case underscores the importance of a comprehensive physical examination, postoperative imaging, and prompt insertion of a lumbar drainage device after spinal surgeries that result in IDs. These findings could aid clinicians in the diagnosis and management of PMs.
